# Clinical spectrum of Chinese hospitalized lung cancer patients with concomitant interstitial lung disease: before and after the new era of LC treatment

**DOI:** 10.1007/s10238-023-00999-1

**Published:** 2023-01-30

**Authors:** Ruxuan Chen, Chi Shao, Xiangning Liu, Hui Huang, Boju Pan, Kai Xu, Rui Zhu, Mei Li, Yang Zhao, Keqi Chen, Mengzhao Wang, Zuojun Xu

**Affiliations:** 1 Department of Pulmonary and Critical Care Medicine, Peking Union Medical College Hospital, Chinese Academy of Medical Sciences and Peking Union Medical College, No.1 Shuaifuyuan Street, Dongcheng District, Beijing, 100730 China; 2 Pathological Department of Anesthesiology, Peking Union Medical College Hospital, Chinese Academy of Medical Sciences and Peking Union Medical College, No.1 Shuaifuyuan Street, Dongcheng District, Beijing, 100730 China; 3 Radiological Department, Peking Union Medical College Hospital, Chinese Academy of Medical Sciences and Peking Union Medical College, No.1 Shuaifuyuan Street, Dongcheng District, Beijing, 100730 China; 4 Medical Records Department, Peking Union Medical College Hospital, Chinese Academy of Medical Sciences and Peking Union Medical College, No.1 Shuaifuyuan Street, Dongcheng District, Beijing, 100730 China

**Keywords:** Interstitial lung disease (ILD), Lung cancer, Underlying causes of ILD, Immunotherapy

## Abstract

This study aimed to explore the general characteristics and spectrum of hospitalized Chinese patients suffering from lung cancer with concomitant interstitial lung disease (LC-ILD). Furthermore, we compared their features before and after the period of immunotherapy for lung cancer. A retrospective analysis of the clinical characteristics of hospitalized LC patients with definite pathological diagnoses was performed from 2014 to 2021. ILD was defined after the review of chest CT imaging. There were 13,085 hospitalized LC patients. Among them, 509 patients (3.89%) had 551 cases of ILD. There were variable underlying causes of ILD, including idiopathic interstitial pneumonia (360 patients), LC treatment-associated ILD (134 cases), and connective tissue disease-associated ILD (55 patients). Although most LC-ILD patients were suffering from adenocarcinoma (204/40.1%), SCLC patients were prone to concomitant ILD (10.8% of all SCLC cases), followed by SCC (9.6% of all SCC cases). All but 10 LC-ILD patients received anti-LC treatment; however, only 39 (10.8%) LC-IIP patients received anti-ILD treatment. There were more LC-ILD patients in the 2018–2021 group than in the 2014–2017 group (5.16% vs. 2.03%, *p* < 0.001). The underlying causes of ILD were significantly different between the 2018–2021 group and the 2014–2017 group (*p* < 0.001). After adjusting for the number of hospitalized patients having the same LC pathological pattern, SCLC was determined to be the most likely to be concomitant with ILD, followed by SCC. Most LC-ILD patients were scheduled for anti-LC therapy; however, treatments for concomitant IIP were usually ignored. LC treatment-associated ILD should receive more attention than before.

## Introduction

With the application of new medications, including targeted therapy, immune therapy, and antifibrotic drugs, the prognoses of lung cancer (LC) and interstitial lung diseases (ILDs) have been greatly improved [1–6]. However, most patients suffering from lung cancer with concomitant interstitial lung disease (LC-ILD) cannot benefit from these novel treatments because of their complicated condition. Therefore, the prognosis of these patients is still poor [7–10].

ILDs are a group of heterogeneous, diffuse parenchymal lung diseases with similar clinical manifestations and disease behaviors but variable causes, including occupational ILD, connective tissue disease (CTD)-ILD, drug-induced ILD, radiation pneumonitis, idiopathic interstitial pneumonia (IIP), etc. [11]. ILDs, especially idiopathic pulmonary fibrosis (IPF), are associated with an increased risk of LC. IPF conveys a fivefold increased risk of LC after adjusting for smoking [12]. The risk of LC was reported to be 3.5- to 7.3-fold higher in ILD patients than in the general population [13].

On the other hand, in the era of precision therapy and immunotherapy, iatrogenic ILDs should never be overlooked. Taxane-containing chemotherapy, ﻿targeted therapies such as epidermal growth factor receptor (EGFR)-﻿tyrosine kinase inhibitors (TKIs), and immune checkpoint inhibitors (ICIs) are common causes of drug-induced ILDs during systemic LC therapies [14–16]. Although targeted therapies and immunotherapy have significantly improved the prognosis of advanced LCs, patients with pre-existing ILD should be treated cautiously because of the increased risk of treatment-induced ILD [17, 18]. Baseline ILD was considered a relative contraindication to ICI therapy [3]. It was recommended to closely monitor LC patients treated with targeted therapy and/or immunotherapy for ILD to reduce the adverse effect on patient outcome through early detection and early management [3, 9].

To elucidate the spectrum of lung cancer with concomitant interstitial lung disease (LC-ILD), we retrospectively summarized a single-center experience in a Chinese tertiary hospital. Furthermore, immune checkpoint inhibitor-related pneumonitis (CIP) is a severe and potentially life-threatening adverse effect of immunotherapy. Most of the severe CIP patients were carefully treated in our institute. Since ICIs were first approved by the China Food and Drug Administration (National Medical Products Administration) for the treatment of LC in 2018, we further compared the features of our hospitalized LC-ILD patients in the 2014–2017 group (LC-ILD patients who were admitted from January 2014 to December 2017) with those in the 2018–2021 group (LC-ILD patients who were admitted from January 2018 to December 2021).

## Materials and methods

### Patients

Pathology-confirmed LC patients (*n* = 13,085) consecutively admitted to Peking Union Medical College Hospital from January 2014 to December 2021 were enrolled in this study. We only included inpatients in the study to ensure the integrity of medical records. Patients receiving TKI therapy and/or radiotherapy were most likely treated at outpatient clinics. These patients were admitted when they developed serious complications. Patients treated with surgical resection, chemotherapy, and/or immunotherapy were all inpatients.

Demographics, clinical features, laboratory tests, pulmonary function tests, and imaging data were extracted from the medical records, which were comprehensively reviewed by two pulmonologists (RX.C. and H.H.). Among these, 509 LC-ILD patients were identified, including both LC patients with pre-existing ILD and those with iatrogenic ILD.

To compare the characteristics of LC-ILD patients before and after the era of immunotherapy, we divided the enrolled subjects into two groups based on admission date: the 2014–2017 group and the 2018–2021 group. In the 2014–2017 group, there were 108 LC-ILD patients among 5316 LC patients. In the 2018–2021 group, there were 401 LC-ILD patients among 7769 LC patients.

### Defining LC, ILD, and emphysema

All LC cases were diagnosed based on pathologic evaluation. We divided LC cases into 5 groups: adenocarcinomas, squamous cell carcinomas (SCCs), small cell lung cancer (SCLC), non-small cell lung neuroendocrine neoplasms (NSCNENs) (including large cell neuroendocrine carcinomas and lung carcinoids), and other non-small cell lung cancer (NSCLC) (e.g., adenosquamous carcinomas, sarcomatoid carcinomas, large cell carcinomas).

ILD was defined by the presence of hallmark manifestations on chest computed tomography (CT) [11]. The classifications of ILD in LC patients included IIPs (further divided into IPF and non-IPF IIPs) and secondary ILDs (e.g., CTD-ILD, occupational ILD, radiation-induced ILD, drug-induced ILD).

**Fig. 1:**
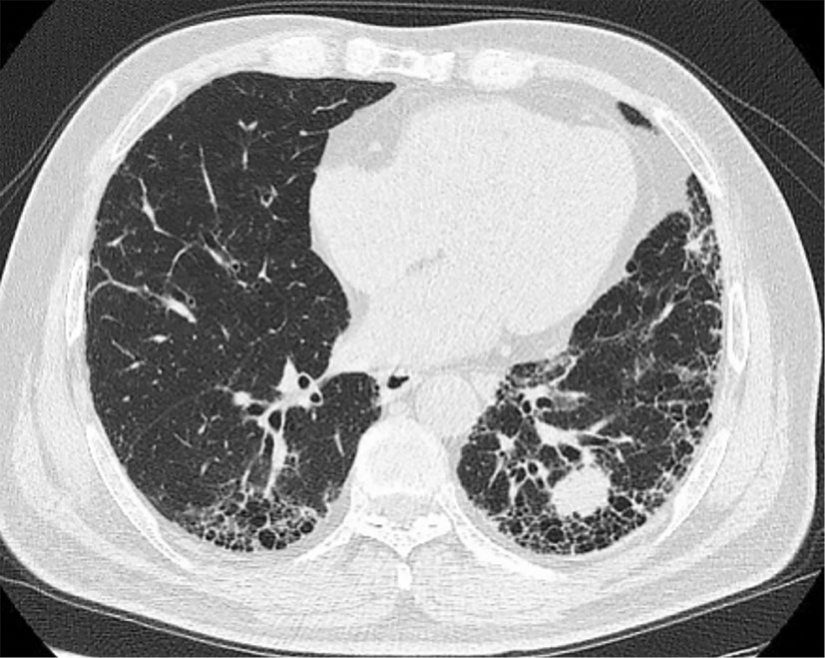
Chest CT for one IPF patient concomitant with small cell lung cancer: chest CT scan showed bilateral ﻿subpleural with basal predominance of the reticulation, interlobular septal thickening, and scattered honeycombs. There was a mass in the lateral basal segment of left inferior lung. He was diagnosed with small cell lung cancer after percutaneous lung biopsy

Emphysema was identified as a region of low attenuation not bounded by visible walls on CT [20]. Chest CT images were reviewed by two pulmonologists (RX.C. and H.H.) and one radiologist (K.X.) in a blinded manner. The typical high-resolution CT (HRCT) findings for LC-ILDs are presented in Fig. 1.

## Results

Demographic and pathological features of enrolled LC patients and LC-ILD patients.

Altogether, 13,085 pathologically diagnosed LC patients were enrolled in this study. There were 5316 LC patients in the 2014–2017 group, 3035 of whom were male (57.1%). The median age was 61 (range 13–95, IQR 53–67) years, and 2869 patients received surgery (54.0%). In the 2018–2021 group, there were 7769 LC patients, 3640 of whom were male (46.9%), which was less than that in 2014–2017 group (46.9% vs. 57.1%, *χ*^2^ = 131.1, *p* < 0.001). The median age was 61 (range 15–94, IQR 53–67) years, and 5473 patients received surgery (54.0%).

The pathological patterns of LC in the 2014–2017 group vs. the 2018–2021 group were recorded as follows: adenocarcinoma 3597/67.7% vs. 6218/80.0%, SCC 839/15.8% vs. 822/10.6%, SCLC 545/10.3% vs. 497/6.4%, NSCNEN 97/1.8% vs. 76/1.0%, and other NSCLCs 238/4.5% vs. 156/2.0%.

**Fig. 2:**
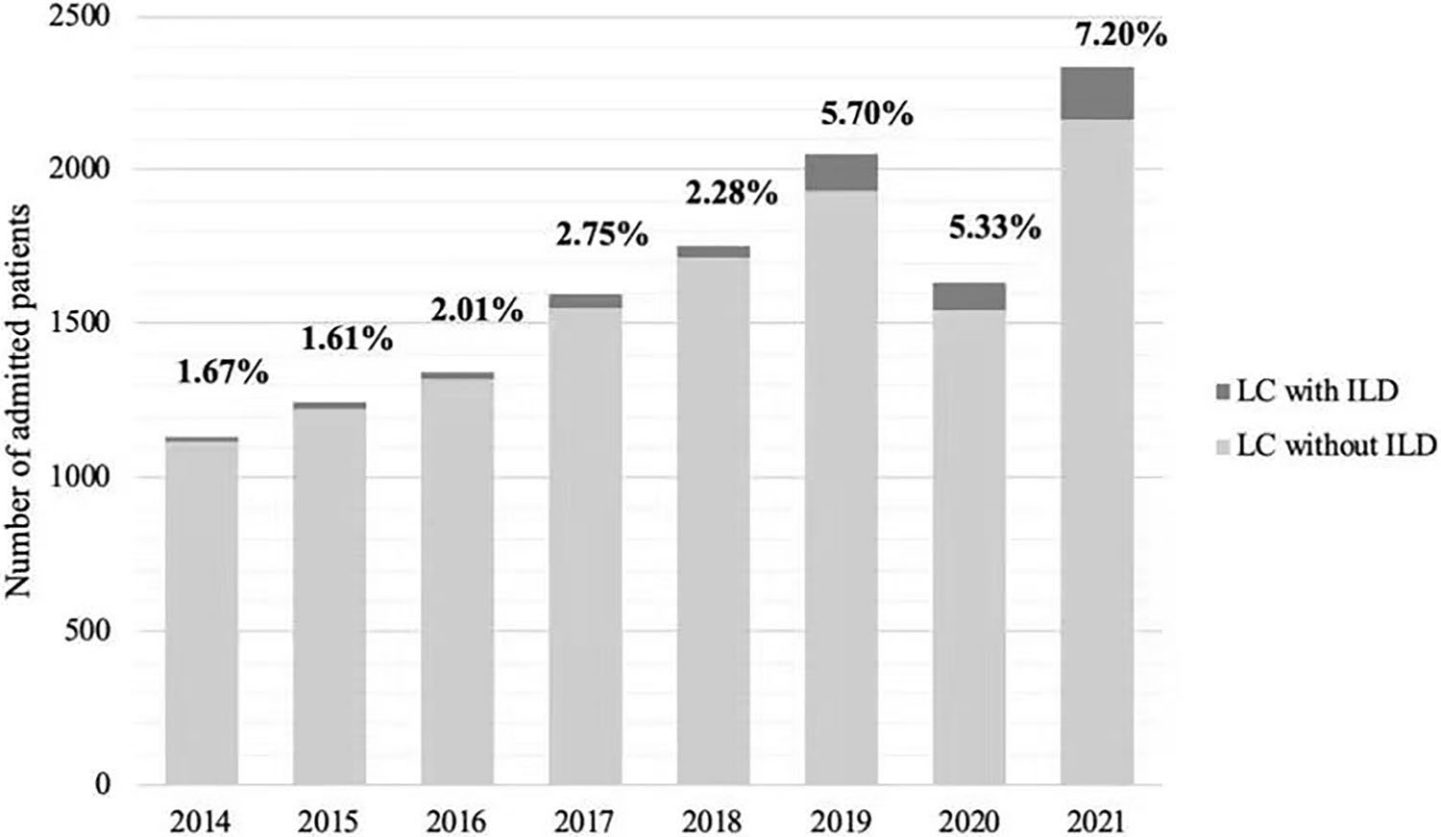
Prevalence of ILD in LC inpatients from 2014 to 2021

Among the 13,085 LC patients, 509 patients (3.89%) had 551 cases of ILD. There were more LC-ILD patients in the 2018–2021 group than in the 2014–2017 group (401/5.16% vs. 108/2.03%, *χ*^2^ = 82.7, *p* < 0.001) (Fig. 2). Among 509 LC-ILD patients, 420 patients were male (82.5%), 399 patients had a smoking history (78.4%), and 231 patients had emphysema (52.8%). There were more male (84.3% vs. 75.9%, p = 0.04) and older (66 vs. 64 years, *p* = 0.01) LC-ILD patients in the 2018–2021 group than in the 2014–2017 group. There were less LC and LC-ILD patients who were admitted in 2020 because the monitor strategy of COVID-19 in China. In Beijing, the COVID-19 patients were treated in designated hospital. LC-ILD patients with COVID-19 were not enrolled in our study.

### The underlying causes of ILD for enrolled LC-ILD patients

**Fig. 3:**
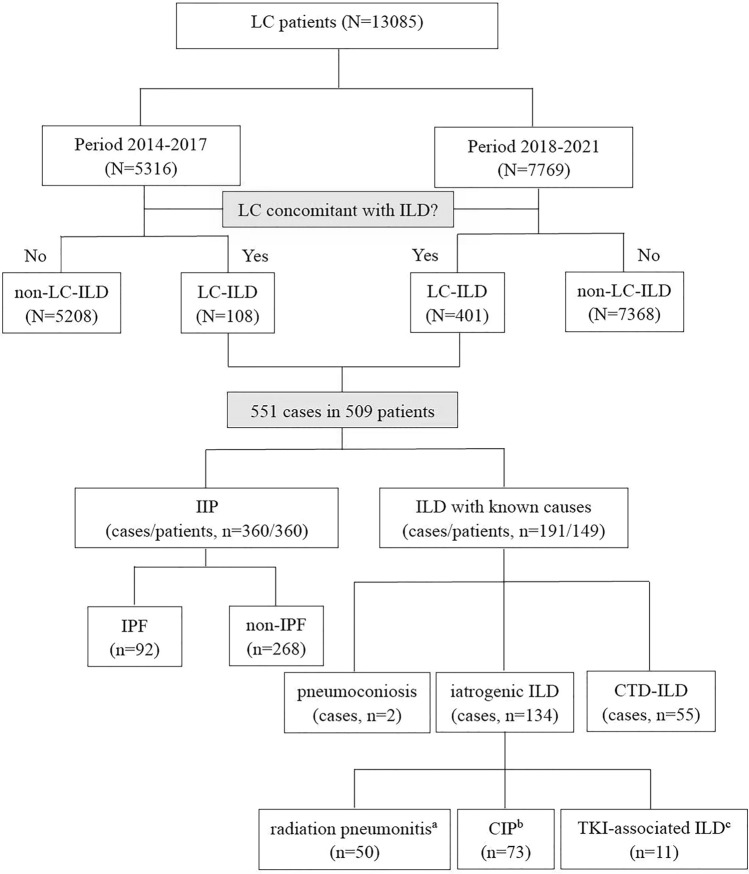
Flow chart of study.** a** Among the 50 cases of radiation pneumonitis, 15 case had pre-existing ILD (13 cases of IIP, 1 case of SSc-ILD, and 1 case of IgG4-RD-ILD), and 11 cases were combined with CIP. **b** Among the 73 cases of CIP, 13 case had pre-existing IIP, and 11 cases were combined with radiation pneumonitis. **c** Among the 11 cases of TKI-associated ILD, 3 cases had pre-existing ILD (2 cases of IIP, and 1 case of RA-ILD)

The detailed study flow is shown in Fig. 3. There were 360 IIP patients in the 509 enrolled LC-ILD patients. Among them, 92 patients had IPF. Twelve IPF patients were prescribed treatment for ILD, with 7 patients receiving N-acetylcysteine, 3 receiving nintedanib, and 2 receiving pirfenidone. Among the non-IPF IIP patients, 27 patients received ILD treatment, with 13 patients taking glucocorticoids, 12 taking N-acetylcysteine, 1 taking nintedanib, and 1 taking pirfenidone. As for LC treatment in the 360 IIP patients, 275 patients had chemotherapy, 66 patients took TKI, 121 patients had immunotherapy (with 80 patients taking immunotherapy as a first-line treatment), 59 patients had radiation therapy, and 8 patients received palliative therapy only. Some of the patients had complications with LC treatment-associated ILDs, with 13 cases suffering from radiation pneumonitis, 13 cases suffering from CIP, and 2 cases suffering from TKI-associated ILD.

Fifty-five CTD-ILD patients were enrolled in the study, and 28 patients were male (50.9%). The mean age was 62.4 ± 9.1 years at the diagnosis of LC. There were 16 cases of rheumatoid arthritis (one of whom later developed TKI-associated ILD), 11 cases of systemic sclerosis (one later developed radiation pneumonitis), 10 cases of inflammatory myopathy, 5 cases of primary Sjögren’s syndrome, 5 cases of antineutrophil cytoplasmic antibody (ANCA)-associated vasculitis, 5 cases of undifferentiated connective tissue disease, 1 case of mixed connective tissue disease, 1 case of systemic lupus erythematosus, and 1 case of IgG4-related disease (who later developed radiation pneumonitis). Most of these patients received treatment for CTD-ILD, with 43 patients taking glucocorticoids and/or immunosuppressants and 2 patients taking pirfenidone. Concerning LC treatment, 33 patients received chemotherapy, 12 patients took TKI, 5 patients had immunotherapy, 7 patients had radiation therapy, and 2 patients only received palliative therapy.

There were 134 cases of LC treatment-associated ILD among 509 patients/551 cases of LC-ILD, including CIP (73 cases), radiation pneumonitis (50 cases), and TKI-associated ILD (11 cases, with 2 cases related to gefitinib, 2 related to almonertinib, 2 related to icotinib, 1 related to osimertinib, 1 related to brigatinib, 1 related to crizotinib, 1 related to furmonertinib, and 1 related to anlotinib). Eight patients (72.7%) with TKI-associated ILD received systemic glucocorticoid treatment. Thirty-seven cases (74%) of radiation pneumonitis were treated with glucocorticoids.

CIP cases have been admitted to our hospital since 2018, and 73 cases were included in this study (8 cases in 2018, 14 cases in 2019, 16 cases in 2020, and 35 cases in 2021). Eleven CIP cases were complicated by radiation pneumonitis, and 13 CIP cases had preexisting IIPs before the administration of immunotherapy. The LC pathological patterns of CIP cases included adenocarcinoma (32 cases), SCC (31 cases), SCLC (7 cases), and other NSCLCs (3 cases).

### The LC characteristics of enrolled LC-ILD patients

**Fig. 4:**
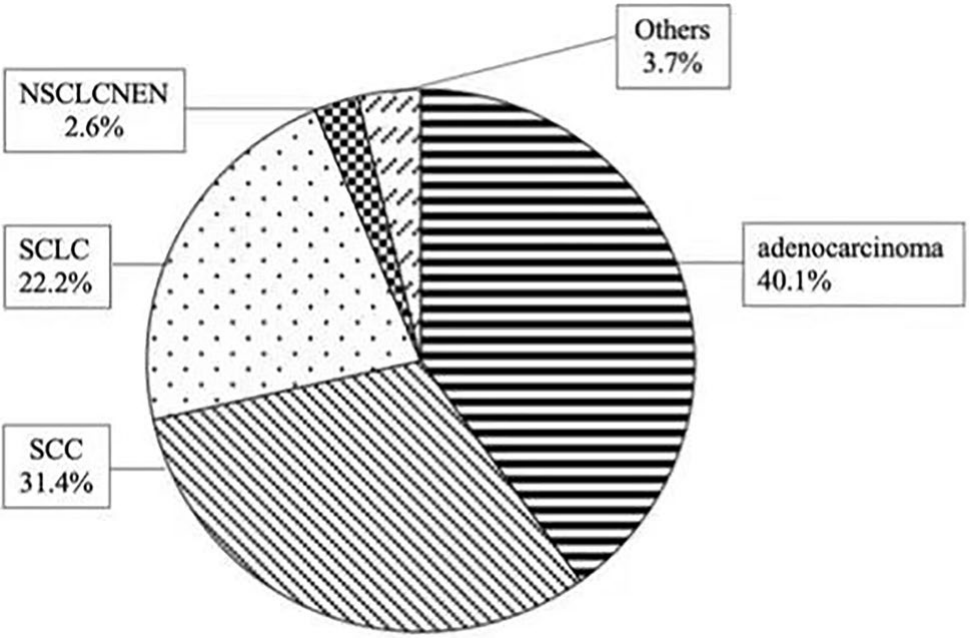
LC pathological patterns of 509 LC-ILD patients

The LC pathological patterns of 509 LC-ILD patients are presented in Fig. 4 and Table 1: adenocarcinoma 204/40.1% (2.1% of all adenocarcinoma cases), SCC 160/31.4% (9.6% of all SCC cases), SCLC 113/22.2% (10.8% of all SCLC cases), NSCNEN 13/2.6% (7.5% of all NSCNEN cases), and other NSCLCs 19/3.7% (4.8% of all other NSCLC cases).

**Table 1:** LC pathological patterns of LC-ILD patients in 2014–2017 and 2018–2021

Pathological patterns	2014–2017	2018–2021
Adenocarcinoma	46 (1.28%)	158 (2.54%)
SCC	33 (3.93%)	127 (15.45%)
SCLC	18 (3.30%)	95 (19.11%)
NSCNEN	3 (3.09%)	10 (13.16%)
Other NSCLCs	8 (3.36%)	11 (7.05%)

All data are presented as number (percentage)

*LC* lung cancer;* ILD* interstitial lung disease;* SCC* squamous cell carcinomas;* SCLC* small cell lung cancer;* NSCNEN* non-small cell lung neuroendocrine neoplasms;* NSCLC*non-small cell lung cancer

### Different characteristics of LC-ILD between 2014–2017 and 2018–2021

A comparison of the clinical and pathological characteristics of LC-ILD patients in the 2014–2017 group and 2018–2021 group is presented in Table 2. Compared with the 2014–2017 group, patients in 2018–2021 had a higher male percentage (84.3% vs. 75.9%, *p* = 0.04), older age (median age 66 years vs. 64 years, *p* = 0.01), fewer IPF cases (15.2% vs. 28.7%, *p* = 0.001), fewer cases who had surgical resection (15.7% vs. 34.3%, *p* < 0.001), and fewer cases suffering from acute exacerbation of ILD after surgical resection (6.3% vs. 10.8%, *p* = 0.045). The differences in the distribution of both the underlying causes of ILD (*p* < 0.001) and the chronological sequences of ILD and LC diagnosis (*p* = 0.003) between the two groups were also statistically significant. No LC-ILD patients who were enrolled in our study were suffered from COVID-19. There were no significant differences of the underlying causes of ILD and the pathological patterns of LC between the 2018–2019 group and 2020–2021 group (Table 3). However, the patients were older in 2020–2021 group than in 2018–2019 group, and more LC-ILD patients were arranged with surgical resection in 2018–2019 group than in 2020–2021 group.

**Table 2:** Different characters of LC-ILD between 2014-2017group and 2018-2021group

Characters	2014–2017^#^	2018–2021^##^	t or* χ*^2^	*p*
Male	82/75.9%	338/84.3%	4.12	0.04
Age[media (IQR)]	64(11)	66 (10)	6.52	0.01
*Smoking* Smoking index[media,(IQR)]	78/72.2% 850(1025)	321/80.0% 800 (475)	3.07	0.08
Emphysema	58/53.7%	211/52.6%	0.04	0.84
*Chronological sequence between the diagnosis of ILD and LC*				
ILD prior to LC	25/23.1%	57/14.2%	11.94	0.003
Simultaneously	70/64.8%	239/59.6%		
LC prior ILD	13/12.0%	105 /26.2%		
*LC pathological pattern* Adenocarcinoma SCC SCLC NSCLCN others	46/42.6% 33/30.6% 18/16.7% 3/2.8% 8/7.4%	158/39.4% 127/31.7% 95/23.7% 10/2.5% 11/2.7%	7.13	0.13
*Causes of ILD*^###^ ILD with underlying causes* IIP**	30/27.8% 84/77.8%	161/40.1% 276/68.8%	5.77	0.02
*ILD with underlying causes*^###^ CTD-ILD Pneumoconiosis Radiation pneumonitis TKI-associated ILD CIP***	20/18.5% 0 9/8.3 1/0.9% 0	35/8.7% 2/0.5% 41/10.2% 10/2.5% 73/18.2%	32.26	< 0.001
*IPF*	31/28.7%	61/15.2%	10.46	0.001
Surgical resection of LC AE-ILD after surgery	37/34.3% 4/10.8%	63/15.7% 4/6.3%	18.54 4.03	< 0.001 0.045

All data are presented as number/% or median (interquartile range)

*LC* lung cancer, ILD *SCC* squamous cell carcinoma,* SCLC* small cell lung cancer;* NSCNEN* non-small cell lung neuroendocrine neoplasms (including large cell neuroendocrine carcinomas and lung carcinoids);* IIP* idiopathic interstitial pneumonia;* CTD-ILD* connective tissue disease associated ILD;* TKI* tyrosine kinase inhibitors;* CIP* immune checkpoint inhibitors related pneumonitis;* IPF* idiopathic pulmonary fibrosis;* AE* acute exacerbation

^#^ There are 114 cases and 108 patients with ILD in 2014–2017 group

^##^ There are 437cases and 401 patients with ILD 2018–2021 group

^###^ Number of ILD cases were applied in these items, otherwise number of ILD patients were applied

*2 cases of radiation pneumonitis and 1 case of TKI-associated ILD had pre-existing CTD-ILD

** 13 cases of radiation pneumonitis, 13 cases of CIP, and 2 cases of TKI-associated ILD had pre-existing IIP

***11 cases of radiation pneumonitis were combined with CIP

**Table 3:** Different characters of LC-ILD between 2018–2019 group and 2020–2021 group

Characters	2018–2019^#^	2020–2021^##^	t or χ^2^	p
Male	123/80.9%	216/86.7%	2.45	0.12
Age[media,(IQR)]	65(60 ~ 70)	67(63 ~ 72)	5.82	0.02
Smoking Smoking index[media,(IQR)]	118/77.6% 600(120 ~ 1000)	198/79.5% 600(300 ~ 1000)	0.2 0.67	0.65 0.42
Emphysema	78/51.3%	133/53.4%	0.17	0.68
*Chronological sequence between the diagnosis of ILD and LC* ILD prior to LC Simultaneously LC prior ILD	25/16.4% 89/58.6% 38/25%	32/12.9% 151/60.6% 66/26.5%	1.01	0.60
*LC pathological pattern* Adenocarcinoma SCC SCLC NSCLCN oOthers	68/44.7% 40/26.3% 35/23.0% 4/2.6% 5/3.3%	90/36.1% 87/34.9% 60/24.2% 8/3.2% 4/1.6%	5.33	0.26
*Causes of ILD*^###^ ILD with underlying causes* IIP**	52/34.2% 107/70.4%	109/43.8% 169/67.9%	3.59 0.28	0.06 0.60
*ILD with underlying causes*^###^ CTD-ILD Pneumoconiosis Radiation pneumonitis TKI-associated ILD CIP***	14/26.9% 2/3.8% 9/17.3% 4/7.7% 23/44.2%	21/19.3% 0/0 32/29.4% 6/5.5% 50/45.9%	7.44	0.11
IPF	20/18.7%	41/24.3%	1.18	0.28
Surgical resection of LC AE-ILD after surgery	31/20.4% 2/6.4%	32/12.9% 2/6.2%	4.06 0	0.04 1

All data are presented as number/% or median (interquartile range)

*LC* lung cancer, ILD* SCC* squamous cell carcinoma,* SCLC* small cell lung cancer;* NSCNEN* non-small cell lung neuroendocrine neoplasms (including large cell neuroendocrine carcinomas and lung carcinoids);* IIP* idiopathic interstitial pneumonia;* CTD-ILD* connective tissue disease associated ILD;* TKI* tyrosine kinase inhibitors;* CIP* immune checkpoint inhibitors related pneumonitis;* IPF* idiopathic pulmonary fibrosis;* AE* acute exacerbation

^#^ There are 159 cases and 152 patients with ILD in 2018–2019 group

^##^ There are 278cases and 249 patients with ILD 2020–2021 group

^###^ Number of ILD cases were applied in these items, otherwise number of ILD patients were applied

* 11cases of radiation pneumonitis and CIP, 2 cases of radiation pneumonitis and 1 case of TKI-associated ILD had pre-existing CTD-ILD

** 13cases of CIP, 7 cases of radiation pneumonitis, and 2 cases of TKI-associated ILD had pre-existing IIP

***1 + cases of radiation pneumonitis were combined with CIP

### Causes of death in LC-ILD patients

Forty LC-IIP patients died during the follow-up: 20 patients died of lung cancer progression, 12 patients died of uncontrolled ILD, 5 patients died of severe pulmonary infection, 2 patients died of severe arrhythmia, and one patient died of immune-related myocarditis. Two LC-CTD-ILD patients died during the follow-up: one patient died of lung cancer progression, and the other died of ILD associated with anti-melanoma differentiation-associated gene 5-positive dermatomyositis.

## Discussion

Most of our hospitalized LC-ILD patients were older males who had a smoking history. Current smoking is a reported risk factor for LC-IPF [21]. In a meta-analysis of IPF and LC, 50–83% of LC-IPF patients were current or former smokers [22], partly because smoking is a common risk factor for both IPF and LC [10, 23]. However, in Gibiot’s study, there was no significant difference in smoking status between LC-ILD patients and LC patients without ILD [10]. Half of our LC-ILD patients had complications with emphysema.

The noniatrogenic ILD and LC in our study were diagnosed simultaneously in 74.3% of the patients. Early diagnosis for LC-ILD is still a great challenge. However, in LC patients with concurrently diagnosed ILD, only a small portion (14.5%) of patients received medications for ILD. Although diagnostic delay was the most common challenge for either ILD or LC management, timely diagnosis and early appropriate management would improve the prognosis of both ILD and LC [24, 25]. However, the clinical manifestations of early ILD or LC are nonspecific and can easily be overlooked by patients and/or generalist respiratory physicians. Long-term or persisting dry cough and/or exertional dyspnea, Velcro rales, decreased diffusion and/or restrictive ventilatory dysfunction in pulmonary function tests, and diffuse lung shadows are common specific characteristics of ILDs [11]. Detailed physical examination, regular chest CT scan, and pulmonary function test should be arranged for patients with persisting cough and/or exertional dyspnea, especially for those with ILD risk factors.

LC treatment-associated ILD has been reported more commonly since the implementation of immunotherapy [26]. In our cohort, more than 20% of LC-ILD cases were associated with LC treatment: CIP was the most common, followed by radiation pneumonitis. CIP should be monitored in LC patients receiving immunotherapy, especially for those with CIP-associated risk factors, including preexisting chronic pulmonary diseases. ILDs [26, 27]. Educating LC patients, their caregivers and CIP first-line physicians would help with the early diagnosis of CIP [28]. Similar to most ILDs, early diagnosis and appropriate management would improve the prognosis of CIP. The difference in causes of ILDs between the 2014–2017 group and the 2018–2021 group was largely attributed to this new type of LC treatment-related ILD.

As adenocarcinoma is the most common LC type in the general population, it is also the most common LC pathological pattern in our LC-ILD cohort, followed by SCC and SCLC. However, compared with the total number of LC patients with the same pathological pattern during the same period, SCLC patients were most likely to have concomitant ILD, followed by SCC. The adenocarcinoma LC patients were the least likely to have ILD. In most previous studies, however, SCC was reported to be the most common LC type in LC-IPF, and adenocarcinoma was the most commonly reported LC type in LC-non-IPF [7, 21, 22]. We noticed that those studies did not take into account the total number of LC patients with the same pathological pattern during the same period. Koyama et al. retrospectively analyzed the prevalence of IIPs in SCLC, and 39.2% of SCLC patients in their study had IIPs, among whom approximately 42.6% had IPF. As immunotherapy has been recommended for treating SCLC, the concurrence of SCLC and ILDs should raise more awareness of this issue with clinicians [29, 30].

In 417 LC patients with noniatrogenic ILD, IIP was the most common cause (360 patients, 92 of whom had IPF), followed by CTD-ILD (55 patients). Most CTD-ILD-LC patients (78.2%) received glucocorticoids and/or immunosuppressants, and a few patients received antifibrotics. However, only a small portion of LC-IIP patients (10.8%) were prescribed treatment for ILD. Naoi et al. reported that antifibrotic medications were associated with a reduced risk of LC development in patients with IPF [31]. Thus, we suggest raising awareness of the importance of ILD treatment in LC-IIP patients and increasing the implementation of antifibrotic treatment in IPF patients [32]. For LC treatments in LC-ILD patients, chemotherapy was the most commonly used treatment in our cohort, and 30% of these patients received immunotherapy (approximately two-thirds of whom had immunotherapy as a first-line regimen). In our cohort, less than 10% (7.8%) of LC-IIP patients were complicated with LC treatment-associated ILD, and the incidence of CIP was 11.4%, which was similar to the reported incidence (13%) in another study [16]. Matsumoto et al. suggested that ICI should be cautiously prescribed for patients with NSCLC with preexisting ILD after they are given a systemic consideration to the risks and benefits of ICI for these patients [33]. In the CTD-ILD-LC patients in our study, approximately 60% received chemotherapy, 10% received immunotherapy, and approximately 5% suffered from LC treatment-associated ILD. For LC patients with CTD, optimized radiation therapy and antiangiogenic therapy are recommended, and immunotherapy might be effective and safe for inactive CTD patients. Furthermore, nintedanib might be a promising medication for CTD-ILD-LC patients [34]. Generally, most of the LC treatment-associated ILD events were controllable in our cohort. Therefore, we suggest that LC-ILD patients should receive adequate LC treatment to improve their prognoses.

There were several limitations in our retrospective study. First, all patients were diagnosed with pathologically confirmed LC and radiologically confirmed ILD, and all of them were admitted to a tertiary hospital; both could lead to selection bias. Second, almost all of our LC patients were not admitted to the ward if they were administered oral TKI tablets or radiation therapy. If their TKI-associated ILD or radiation pneumonitis was not severe, they were treated in an outpatient clinic and thus not included in our study. Third, our study was a retrospective study, and the clinical features and therapy regimens varied from patient to patient. Fourth, the patients who were suffered from COVID-19 were admitted in designated hospitals in Beijing, so the number of LC patients and ILD-LC patients was decreased in 2020. A multicenter, well-designed prospective study is expected in the future.

## Conclusions

After adjusting for the number of hospitalized patients having the same LC pathological pattern, SCLC was determined to be the most likely to be concomitant with ILD, followed by SCC. Most LC-ILD patients were scheduled for anti-LC therapy; however, treatments for concomitant IIP were usually ignored. As the spectrum of ILD in LC-ILD has changed significantly in the era of immunotherapy, LC treatment-associated ILD should receive more attention than before.

## Data Availability

All data generated or analyzed during this study are included in this published article. Besides, any additional data/files may be obtained from the corresponding author on reasonable request.
